# Characterization of Clade 2.3.2.1 H5N1 Highly Pathogenic Avian Influenza Viruses Isolated from Wild Birds (Mandarin Duck and Eurasian Eagle Owl) in 2010 in Korea

**DOI:** 10.3390/v5041153

**Published:** 2013-04-23

**Authors:** Jun-Gu Choi, Hyun-Mi Kang, Woo-Jin Jeon, Kang-Seuk Choi, Kwang-Il Kim, Byung Min Song, Hee-Soo Lee, Jae-Hong Kim, Youn-Jeong Lee

**Affiliations:** 1 Animal, Plant and Fisheries Quarantine and Inspection Agency, 175 Anyangro, Manangu, Anyang, Gyeonggi, 430-757, Republic of Korea; E-Mails: happythomas@korea.kr (J.-G.C.); greenkang@korea.kr (H.-M.K.); jeonwj@korea.kr (W.-J.J.); kchoi0608@korea.kr (K.-S.C.); kimki@korea.kr (K.-I.K.); todays1@korea.kr (B.M.S.); leehs0415@korea.kr (H.-S.L.); 2 College of Veterinary Medicine, Seoul National University, 599 Gwanakro, Seoul, 151-742, Republic of Korea

**Keywords:** H5N1, highly pathogenic avian influenza, clade 2.3.2.1

## Abstract

Starting in late November 2010, the H5N1 highly pathogenic avian influenza (HPAI) virus was isolated from many types of wild ducks and raptors and was subsequently isolated from poultry in Korea. We assessed the genetic and pathogenic properties of the HPAI viruses isolated from a fecal sample from a mandarin duck and a dead Eurasian eagle owl, the most affected wild bird species during the 2010/2011 HPAI outbreak in Korea. These viruses have similar genetic backgrounds and exhibited the highest genetic similarity with recent Eurasian clade 2.3.2.1 HPAI viruses. In animal inoculation experiments, regardless of their originating hosts, the two Korean isolates produced highly pathogenic characteristics in chickens, ducks and mice without pre-adaptation. These results raise concerns about veterinary and public health. Surveillance of wild birds could provide a good early warning signal for possible HPAI infection in poultry as well as in humans.

## 1. Introduction

Highly pathogenic avian influenza (HPAI) is one of the most devastating poultry diseases. The H5N1 subtype HPAI virus has become widely distributed, especially in Asia, Europe and Africa since 2003 [1]. Some countries have applied early detection and rapid response measures to control HPAI, including destroying infected birds, and have successfully eradicated the disease. However, the virus is still circulating and evolving in several Asian countries and Africa [2,3,4,5,6]. In response to the rapidly evolving Eurasian H5N1 HPAI viruses, the WHO/OIE/FAO Evolution Working Group designed a nomenclature system and has classified the viruses according to the phylogenetic characterization and nucleotide sequence divergence of the hemagglutinin (HA) genes [7]. Since the ancestor virus was identified in 1996 in China [8], a total of 10 genotypes (Clade 0–9) of H5N1 HPAI viruses have been isolated and have been further diverged into second-, third- and fourth-order clades. Some of the genotypes are still circulating and evolving, while others have been replaced by novel genetic variants [7].

Aquatic wild birds, especially migratory ducks, are believed to be the natural reservoirs of all 16 HA and nine neuraminidase (NA) subtypes of avian influenza viruses and are asymptomatic [9]. However, many types of wild water fowl died of the H5N1 HPAI virus infection in Hong Kong in 2002 [10], and significant numbers of wild birds have been affected in several countries since 2005 [11,12,13,14]. 

Since 2003 in Korea, there have been four H5N1 HPAI outbreaks caused by different virus genotypes (clade 2.5, 2.2 and 2.3.2.1), and large numbers of commercial poultry such as chickens and ducks have been destroyed to try to halt the outbreaks [15,16,17]. Before 2010, there have been three H5N1 HPAI outbreaks in Korea, and although it was strongly suspected that migratory birds participated in the transmission of the viruses to poultry flocks based on epidemiological data, dead or clinically ill wild migratory birds were not found. However, in the outbreak that occurred during the winter season of 2010/2011, unexpectedly high infection and mortality rates caused by H5N1 HPAI virus were recorded in wild birds such as wild ducks, including mandarin ducks (*Aix galericulata*), mallards (*Anas platyrhynchos*) and Baikal teals (*Anas formosa*), and in raptors, including the Eurasian eagle owl (*Bubo bubo*), Eurasian sparrowhawk (*Accipiter nisus*) and common kestrel (*Falco tinnunculus*) [18,19,20]. 

To investigate the genetic and pathogenic properties of the recent Korean H5N1 HPAI viruses according to the hosts from which the viruses were isolated, we chose two H5N1 HPAI viruses isolated from a mandarin duck (Anseriformes) and an Eurasian eagle owl (Strigiformes), which were the most affected wild bird species in the 2010/2011 Korean HPAI outbreaks [18]. This study analyzed the genetic and phylogenetic characteristics of the two isolates, and assessed their pathogenicity in chickens, ducks and mice.

## 2. Results

### 2.1. Virus Isolation

A/Eurasian eagle owl/Korea/23/2010 was isolated from an ill Eurasian eagle owl discovered on 26 November 2010 on a hill near Cheonsu Bay, which is located in the mid-region of the western coast of South Korea. This region is one of the largest wild bird habitats in Korea. At the time of rescue, the owl was severely ill, and it died the next day. The dead body was submitted to the Animal, Plant and Fisheries Quarantine and Inspection Agency (QIA), and H5N1 HPAI virus was isolated from the brain and proventriculus. A/mandarin duck/Korea/PSC24-24/2010 was isolated from wild bird fecal samples collected at Pung-se stream on 28 December 2010, located in Cheonan City, Chungnam Province, Korea. This site was only 4 km from the site of the first recorded 2010 HPAI case in poultry, on a duck farm on 29 December 2010. 

### 2.2. The Disease Situation on Duck Farms during 2010/2011 H5N1 HPAI Outbreak in Korea

During the 2010/2011 winter season, wild birds affected by H5N1 HPAI viruses were found in Korea, and subsequently, HPAI broke out in domestic poultry [18]. A total of 53 poultry flocks were diagnosed as H5N1 HPAI. Among them, 33 cases (62.3%) occurred in duck farms (10 breeder duck farms and 23 meat-type duck farms). Although the primary clinical signs in the Galliformes birds (chicken, pheasant and quail) were the steeply increased mortality, which was independent of age of animals, there was evident age-dependence in the pathogenicity in ducks. The most prominent clinical sign in under 5-week-old meat type ducks was the high mortality of up to 31.0% before diagnosis and culling, while those of breeder ducks over 18 weeks of age were reduced feed consumption and egg production (~80.0%). Affected breeder ducks rarely died (Table 1). 

### 2.3. Genetic and Phylogenetic Analysis

**Figure 1:**
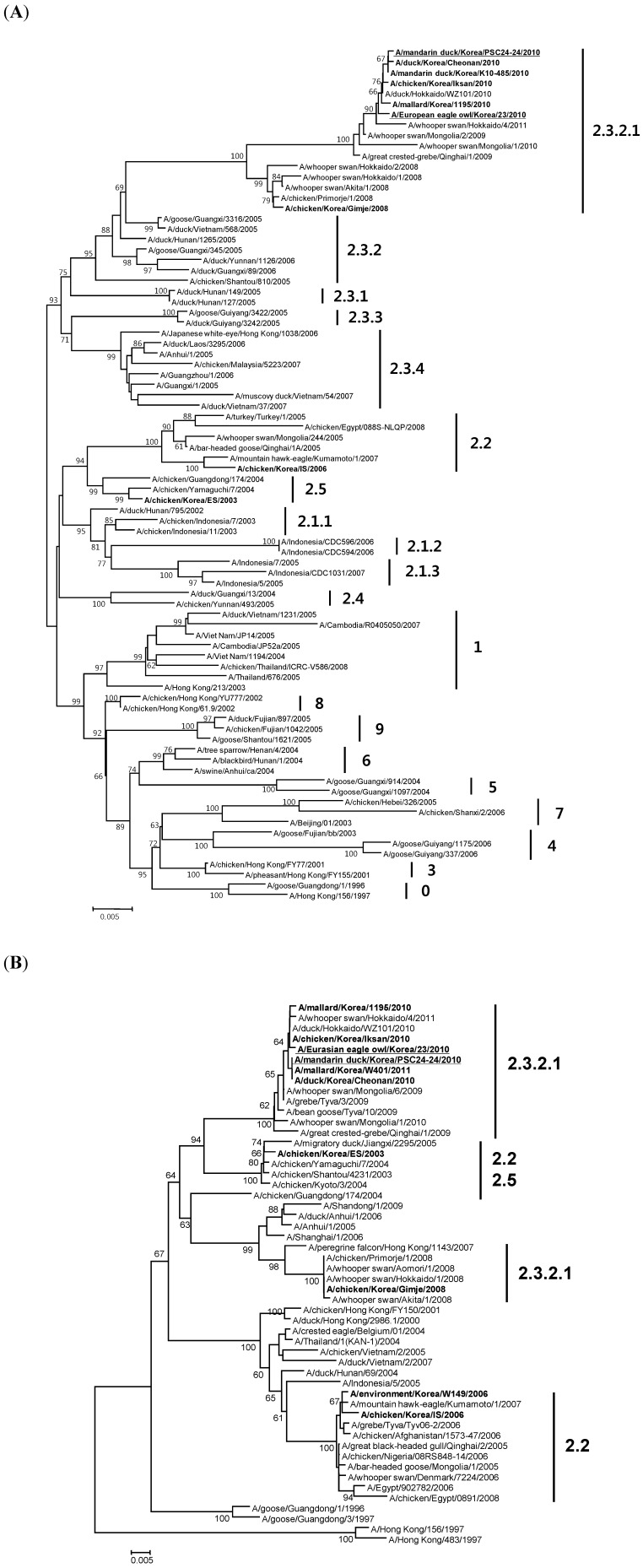
Phylogenetic analysis of the (**A**) hemagglutinin (HA) and (**B**) acidic polymerase (PA) genes. The Korean H5N1 HPAI viruses are indicated in bold, and the two isolates analyzed in this study are underlined. The genotypes (clade) of the viruses are indicated with vertical lines. The sequences were multiply aligned, and the phylogenetic tree was constructed with the neighbor-joining method (bootstrap value of 1000) using MEGA5 software. The scale bar indicates the average number of nucleotide substitutions per site.

All eight genes of the two Korean wild bird origin viruses were demonstrated to be phylogenetically closely related to the clade 2.3.2.1 viruses that were isolated in 2009–2011 from Korea, China, Mongolia, Russia and Japan (Fig. 1 (A)) [13,14,20,21,22,23]. Their acidic polymerase (PA) genes were phylogenetically distinguished from the 2008 Korean (A/chicken/Korea/Gimje/2008) and Japanese (A/whooper swan/Hokkaido/2/2008) isolates, which were classified into the same genetic group (clade 2.3.2.1). Instead, the PA genes were related to those of the HPAI viruses isolated in Korea (A/chicken/Korea/ES/2003), Japan (A/chicken/Yamaguchi/7/2004) and south China (A/chicken/Shantou/4231/2003) in 2003 and 2004 (Fig. 1 (B)). The deduced amino acid sequences of the HA cleavage site of the two presently isolated H5 HPAI viruses revealed a typical highly pathogenic motif (PQRERRRKR/GLF), and the amino acid residues on the receptor binding sites (RBS) were 226 glutamine (Q) and 228 glycine (G) (H3 numbering), which are characteristic of avian influenza virus. Both viruses possessed a 20 amino acid deletion in the NA stalk region. Instead of 224 proline (P) and 383 aspartic acid (D), which have been proposed to be a virulence marker in ducks [24], the corresponding amino acids of the PA protein of the two isolates were 224 serine (S) and 383 D. The NS1 protein displayed an avian origin PDZ motif (ESEV) and a five-amino-acid deletion at the C-terminal region. 

When comparing the amino acid sequences of the two isolates, differences in 10 amino acids were evident between the PSC24-24 and EEO/23 viruses [three in PB2: glutamic acid (E) 69 G, isoleucine (I) 338 valine (V) and S 590 G); one in PA: (I 178 V); four in HA: (tyrosine (Y) 136 D, D 140 E, E 200 lysine (K) and K 509 (E); one in NA (K 291 E); and one in NS1 (leucine (L) 142 I)].

### 2.4. Virus Replication in Experimentally Inoculated Shickens, Ducks and Mice

#### 2.4.1. Chicken noculation experiments

To determine the pathogenicity in chickens, 1/10 diluted infectious allantoic fluid of the PSC24-24 (10^7.2^ EID_50_/0.1 ml) and EEO/23 (10^6.5^ EID_50_/0.1 ml) viruses were intravenously inoculated into eight 6-week-old chickens according to OIE regulations. The mean death time (MDT) of the PSC24-24 and EEO/23 were 58.2 h and 44.1 h, respectively, and the intravenous pathogenicity indices (IVPI) of the PSC24-24 and EEO/23 in chickens were 2.74 and 2.86, respectively, classifying the viruses as a HPAI virus according to OIE criteria. In chickens inoculated intranasally with 10^6.0^ EID_50_/0.1 ml of EEO/23 virus, the MDT was recorded as 75 h, and the virus titers of the tissue samples taken from dead chickens ranged from 10^4.7^ to 10^5.8^ TCID_50_/0.1 ml (Table 2).

#### 2.4.2. Duck inoculation experiments

**Figure 2:**
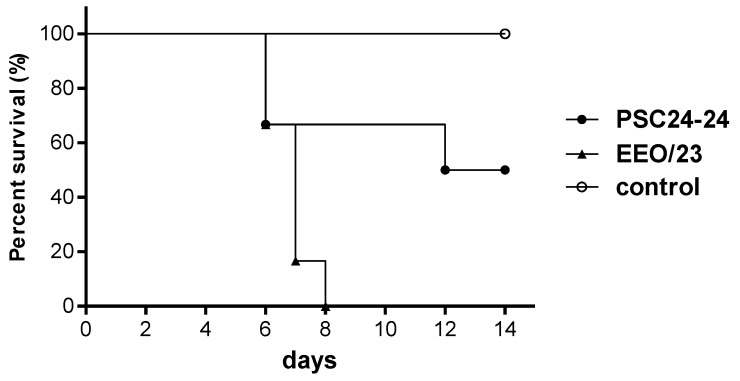
Survival curves of the experimentally inoculated ducks. Six 2-week-old ducks were inoculated with 10^6.0 ^EID_50_/0.1 ml of A/mandarin duck/Korea/PSC24-24/2010 (PSC24-24) and A/Eurasian eagle owl/Korea/23/2010 (EEO/23), respectively. When comparing the survival curves of the two viruses, although they showed different mortality rates of 50% (PSC24-24) and 100% (EEO/23), there was no significant difference between the two viruses (log-rank test, *p* = 0.0538).

To evaluate the pathogenicity in ducks, eight ducks were intranasally inoculated with PSC24-24 or EEO/23 virus, and after 8 h, three other ducks were co-housed with the infected ducks as a contact group. Among the eight inoculated ducks, 2 ducks in each virus inoculated group were sacrificed for virus recovery in various tissues. Three of the remained six ducks inoculated with the PSC24-24 virus died; two died at 6 days post-inoculation (dpi) and the other died at 12 dpi, producing a MDT of 8.0 days. One of the three ducks in the PSC24-24 contact group died at 9 dpi. All remained six ducks inoculated with the EEO/23 virus died within 8 dpi (the MDT was 6.8 days), and one of the three ducks in the EEO/23 contact group died at 9 dpi (one duck in this contact group died accidentally at 2 dpi.). However, when we compared the survival curves using the log-rank test, no statistical significance was observed between the PSC24-24- and the EEO/23-infected ducks (*p* = 0.0538, Fig. 2). During the experiments, the ducks in each virus inoculated group showed clinical signs from 5 dpi, and some displayed symptoms of conjunctivitis, corneal opacity and torticollis.

In the PSC24-24-inoculated group, virus was recovered from oropharyngeal (OP) and cloacal (CL) swab samples until 10 dpi. The virus peaked at 3 dpi for OP swabs (10^2.8^ TCID_50_/0.1 ml) and 7 dpi for CL swabs (10^2.4^ TCID_50_/0.1 ml). The EEO/23 virus was detected in OP and CL swabs of the inoculated ducks until 7 dpi (all six ducks had died by this time). Virus peaked at 3 dpi for OP swabs (10^2.6^ TCID_50_/0.1 ml) and 5 dpi for CL swabs (10^1.4^ TCID_50_/0.1 ml) (Table 3). In the contact groups of the two viruses, the viruses were recovered from 3 dpi until 7–10 dpi in the OP samples and from 5 dpi to 7–10 dpi in the CL samples. 

Both of the viruses replicated systemically in ducks, as can be observed by the recovery of virus from various tissues, including the brain (Table 4). Ducks sacrificed at 3 dpi did not show apparent clinical signs, yet a significant amount of virus (mean titer up to 10^5.6^ TCID_50_/0.1 ml) was recovered from all tested tissues. The titers of the PSC24-24 and EEO/23 isolates from the dead ducks in the inoculated group were recorded as 10^5.2^ and 10^5.0^ TCID_50_/0.1 ml, respectively, in the brain and 10^4.1^ and 10^5.1^ TCID_50_/0.1 ml, respectively, in the lung. However, one dead duck in both of the contact groups showed relatively lower virus titers in tissues compared to those of the virus-inoculated ducks. The serum antibody level of the surviving ducks was 2^5^ – 2^7^ hemagglutination inhibition units (Table 3).

#### 2.4.3. Mouse inoculation experiments

**Figure 3:**
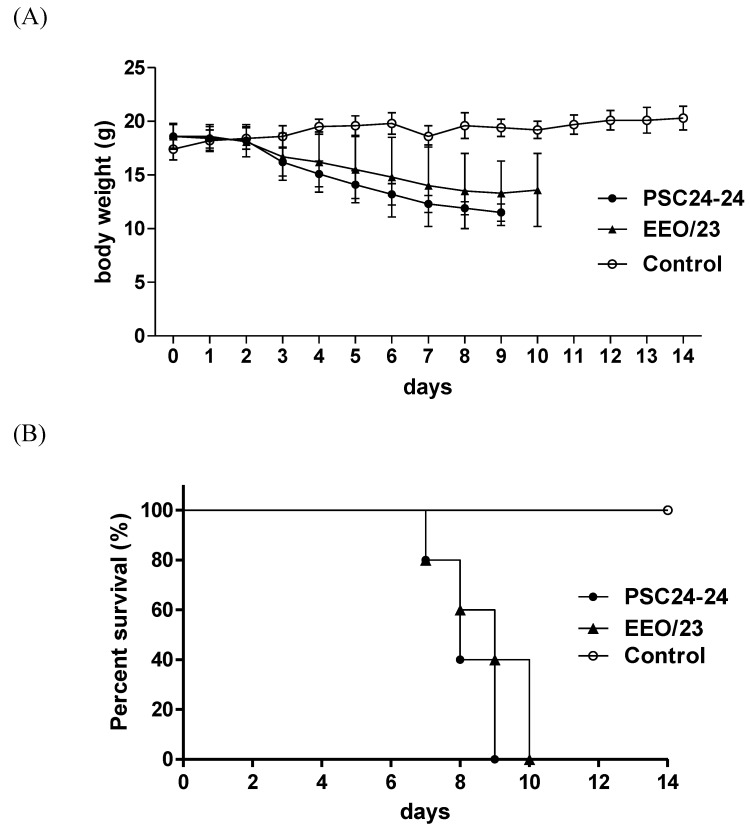
Body weight changes and survival curves of mice.Five mice were inoculated with 10^6.0 ^EID_50_/50 μl of A/mandarin duck/Korea/PSC24-24/2010 (PSC24-24) and A/Eurasian eagle owl/Korea/23/2010 (EEO/23), respectively, and observed for 14 days. (**A**) When comparing the body weight of the mice, the weight of the PSC24-24-inoculated group was significantly different from that of the control group after 3 dpi and that of the EEO/23 group was significantly different after 4 dpi (ANOVA, p<0.05). (**B**) All 5 of the mice in each group died within 10 days. When comparing the survival curves of the two viruses, there was no significant difference betweenthe two viruses (log-rank test, *p=*0.2615).

To determine the pathogenic potential of the two Korean HPAI viruses in mammals, 10^6.0 ^EID_50_/50 μl of each virus was used to inoculate five 6-week-old female BALB/c mice. The infected mice displayed severe clinical signs, including ruffled fur, depression, labored breathing and severe weight loss (Fig. 3A). All five mice in either group died within 10 dpi, and there was no statistical significance in the survival curves (Fig. 3B; log-rank test, *p* = 0.2615). The PSC24-24 and EEO/23 viruses displayed a similar MLD_50_ (10^3.4^ and 10^3.6^ EID_50_, respectively). However, the EEO/23 virus produced a slightly lower MID_50_ (10^1.5^ EID_50_) than PSC24-24 (10^2.3^ EID_50_) (Table 5). To elucidate the virus replication in various tissues, we collected multiple organs such as brain, lung, liver, spleen and kidney from three mice inoculated with 10^6.0 ^EID_50_ of each virus at 3 and 6 dpi and titrated this replication in chicken embryo fibroblasts. The inoculated viruses were recovered from the lung (mean virus titers, 10^5.7^ TCID_50_/0.1 ml for both viruses) and spleen (10^2.4^ TCID_50_/0.1 ml for PSC24-24 and 10^1.2^ TCID_50_/0.1 ml for EEO/23), but not from the brain, liver and kidney collected at 3 dpi. However, viruses were recovered from the brain (10^3.4^ and 10^2.8^ TCID_50_/0.1 ml for PSC24-24 and EEO/23, respectively) and lung (10^4.5^ and 10^3.8^ TCID_50_/0.1 ml for PSC24-24 and EEO/23, respectively)collected at 6 dpi (Table 5).

**Table 1:** Disease situation of selected highly pathogenic avian influenza (HPAI) virus-infected domestic duck farms during the 2010/2011 H5N1 HPAI outbreak in Korea.

Duck type	Farm location^a^	Age (weeks)	Flock size	Clinical signs	Mortality^b^ (%)	Remarks
Breeder duck	Cheonan, Chungnam	36	10,700	Greenish diarrhea, Reduced egg production (61%)	-	Index farm
				Reduced feed consumption (20%)		
	Anseong, Gyeonggi	70/88	45,000	Suppression, Reduced egg production (80%)	-	
	Cheonan, Chungnam	66	13,360	Greenish diarrhea, Reduced egg production (50%)	0.1%	
				Reduced feed consumption (20%)		
	Icheon, Gyeonggi	52/56	8,300	Suppression, Reduced egg production (over 70%)	Rare death	
	Hwaseong, Gyeonggi	18	8,000	Reduced feed consumption, Greenish diarrhea	-	
Meat type duck	Yeongam, Jeonnam	5	14,500	Suppression, Increased mortality	31.0	
	Yeongam, Jeonnam	5	10,600	Suppression, Increased mortality	4.7	
	Yeongam, Jeonnam	3	20,000	Suppression, Increased mortality	7.9	
	Naju, Jeonnam	4	15,000	Suppression, Increased mortality	8.0	
	Yeongam, Jeonnam	5	11,200	Suppression, Increased mortality	14.6	
	Naju, Jeonnam	4	5,000	Torticollis, Leg paralysis, Greenish diarrhea	> 2.7	
				Increased mortality		
	Damyang, Jeonnam	1	13,300	Suppression, Diarrhea, Cyanosis of beak	17.3	

^a^ City, Province ^b^ The mortality is the ratio of dead ducks to the number of animals in the farm until the affected animals were destroyed.

**Table 2:** Pathogenicity and virus distribution of the Korean H5N1 HPAI viruses in chickens.

Virus	Inoculation route	Virus titer (log_10_ TCID_50_/0.1 ml, mean ± standard deviation)								MDT^a ^(hours)	IVPI^b^
		Brain	Trachea	Lung	Spleen	Kidney	Heart	Muscle	Cecal tonsil		
PSC24-24	Intravenous	nt	nt	nt	nt	nt	nt	nt	nt	58.2	2.74
EEO/23	Intravenous	nt	nt	nt	nt	nt	nt	nt	nt	44.1	2.86
	Intranasal	5.1 ± 0.5	5.2 ± 0.5	5.8 ± 0.7	5.0 ± 0.7	5.2 ± 0.8	5.6 ± 0.7	4.7 ± 0.8	4.9 ± 0.9	75	na

A/Eurasian eagle owl/Korea/23/2010 (EEO/23) was inoculated in eight 6-week-old SPF chickens via intravenous (10^6.5 ^EID_50_/0.1 ml) and intranasal routes (10^6.0^ EID_50_/0.1 ml). The viral titers of the tissues (10% homogenates) from the dead chickens in the intranasally inoculated group were measured in CEF. The detection limit of the CEF cell culture was set to 10^0.6^ TCID_50_/0.1 ml. ^a ^MDT: mean death time ^b ^IVPI: intravenous pathogenicity index nt: not tested, na: not applicable

**Table 3:** Virus re-isolation with swab samples from experimentally inoculated ducks. Eight ducks were inoculated intranasally with 10^6.0 ^EID_50_/0.1 ml of A/mandarin duck/Korea/PSC24-24/2010 (PSC24-24) and A/Eurasian eagle owl/Korea/23/2010 (EEO/23), and 3 ducks were co-housed with the infected ducks as a contact group. Oropharyngeal and cloacal swab samples were collected on the indicated day, and the virus was titrated in CEF. Virus titer is the average of the calculable positive samples. The detection limit of the CEF cell culture was set to 10^0.6^ TCID_50_/0.1 ml.

Virus	Group	Sample	virus titer (log_10_ TCID_50_/0.1 ml, mean±standard deviation)						HI titer ^c^
			1dpi	3dpi	5dpi	7dpi	10dpi	14dpi	
PSC24-24	Inoculated	OP	1.6±0.1 (4/8) ^a^	2.8±0.7 (8/8)	2.6±0.8 (6/6)	+ (2/4)	- (0/4)	- (0/3)	5.3±0.6
		CL	- (0/8)	1.9±1.1 (6/8)	1.8±0.9 (4/6)	2.4±1.0 (3/4)	0.9 (1/4)	- (0/3)	
	Contact	OP	- (0/3)	2.2±0.6 (2/3)	2.1±0.4 (3/3)	2.0±1.4 (3/3)	- (0/2)	- (0/2)	5.0±0.0
		CL	- (0/3)	- (0/3)	1.4±0.1 (2/3)	1.1±0.8 (3/3)	0.6 (1/2)	- (0/2)	
EEO/23	Inoculated	OP	+ (2/8)	2.6±0.6 (8/8)	2.6±1.7 (5/6)	1.9±1.0 (4/4)	nt	nt	nt
		CL	+ (3/8)	1.2±0.8 (5/8)	1.4±0.7 (6/6)	- (0/4)	nt	nt	
	Contact ^b^	OP	- (0/3)	2.5 (1/2)	1.6 (2/2)	0.8 (2/2)	+ (1/1)	- (0/1)	7.0
		CL	- (0/3)	- (0/2)	+ (2/2)	2.5 (1/2)	- (0/1)	- (0/1)	

^a^ Number of virus isolation / number of tested samples ^b^ One of the three ducks accidentally died at 2 dpi, but its data are not presented in the table. ^c^ HI (log_2_) titer of serum samples collected from the survived ducks at 14 dpi. OP: oropharyngeal swab sample; CL: cloacal swab sample; -: virus was not detected; +: virus was detected but the titer was not calculable; nt: not tested.

**Table 4:** Replication of the H5N1 AIVs in tissues of the ducks. 10^6.0 ^EID_50_/0.1 ml of A/mandarin duck/Korea/PSC24-24/2010 (PSC24-24) and A/Eurasian eagle owl/Korea/23/2010 (EEO/23) were inoculated intranasally to eight 2-week-old ducks, and 3 ducks were co-housed with the infected ducks as a contact group. Virus replication in tissues from sacrificed and dead birds in the inoculated groups were measured in CEF with 10% tissue homogenates. Virus titer is the average of calculable positive samples. The detection limit of the CEF cell culture was set to 10^0.6^ TCID_50_/0.1 ml.

Virus	Route	Animal status	Number of tested	Virus titer (log_10_ TCID_50_/0.1 ml, mean ± standard deviation)							
				Brain	Trachea	Lung	Spleen	Kidney	Heart	Muscle	Cecal tonsil
PSC24-24	Inoculated	Dead^ a^	3	5.2 ± 0.5	3.5 ± 1.0	4.1 ± 0.6	-	4.0 ± 0.8	4.1 ± 2.1	4.2	3.4
		Sacrificed ^b^	2	3.1 ± 0.4	5.6 ± 0.1	4.6 ± 0.1	3.4 ± 0.8	4.0 ± 0.5	4.5 ± 1.1	3.4 ± 1.1	2.4 ± 0.2
	Contact	Dead^ c^	1	2.6	2.5	3.6	1.6	3.5	4.5	-	2.6
EEO/23	Inoculated	Dead ^d^	6	5.0 ± 1.2	4.0 ± 2.2	5.1 ± 1.0	4.3 ± 2.0	4.3 ± 0.9	3.7 ± 1.3	3.0 ± 0.3	3.9 ± 1.0
		Sacrificed ^b^	2	2.6	3.0 ± 0.3	3.6 ± 0.1	2.7 ± 0.0	2.5 ± 0.1	1.8 ± 0.4	+	2.4
	Contact	Dead^ c, e^	1	1.6	+	+	-	2.3	2.5	1.6	1.5

^a^ Two ducks died at 6 dpi and one duck at 12 dpi. The duck that died at 12 dpi was diagnosed with colibacillosis, and the virus was not recovered from all of the tested tissues. ^b^ Two ducks were sacrificed at 3 dpi ^c^ One duck died at 9 dpi in both of the two groups. ^d^ Two, 3 and 1 ducks died at 6, 7 and 8 dpi, respectively. ^e^ One of the three ducks accidentally died at 2 dpi, and its data are not included in the table. -: virus was not detected; +: virus was detected but the titer was not calculable.

**Table 5:** Virus distribution in experimentally inoculated mice.

Virus	dpi	Virus titer (log_10_ TCID_50_/0.1 ml, mean ± standard deviation)					MLD_50_ (EID_50_)	MID_50_ (EID_50_)
		Brain	Lung	Liver	Spleen	Kidney		
PSC24-24	3dpi	-	5.7±0.1	-	2.4±0.9	-	10^3.4^	10^2.3^
	6dpi	3.4±1.1	4.5±0.1	-	+	-		
EEO/23	3dpi	-	5.7±0.4	-	1.2^b^	-	10^3.6^	10^1.5^
	6dpi	2.8^a^	3.8±0.0^c^	-	-	-		

Six mice were inoculated with 10^6.0 ^EID_50_/50 μl of A/mandarin duck/Korea/PSC24-24/2010 (PSC2424) or A/Eurasian eagle owl/Korea/23/2010 (EEO/23). Three mice were sacrificed on the indicated day, and organs were taken to determine the virus replication in the tissues. The virus titer was measured with 10% tissue homogenates. For assessing the mouse LD_50 _(MLD_50_), 10-fold serially diluted viruses were inoculated and observed for 14 days. For the mouse ID_50_ (MID_50_), lung tissues from 3 inoculated mice per group were collected at 3 dpi. The virus titer is presented as the average of the calculable positive samples. The detection limit of the CEF cell culture was set to 10^0.6^ TCID_50_/0.1 ml. ^a^ One of 3 mice was virus positive. ^b^ Three of 3 mice were virus positive, but the titers from two mice were not calculable. ^c^ Two of 3 mice were virus positive. +: virus was detected, but the titer was not calculable.; -: virus was not detected.

## 3. Discussion

In Korea during the 2010/2011 winter season, an outbreak of H5N1 HPAI involved 53 poultry farms (18 chicken, 33 duck, one quail and one pheasant farm). Prior to this outbreak, H5N1 HPAI viruses were detected in wild birds, with 20 cases of HPAI recognized in various wild bird species. Among them, mandarin ducks and Eurasian eagle owls were the wild birds that were the most affected by the H5N1 HPAI virus [18]. 

In this study, we chose two wild bird origin viruses, PSC24-24 and EEO/23, which were isolated from a fecal sample of a wild duck (mandarin duck) and a dead *Strigiformes* bird (Eurasian eagle owl), respectively. Although the direct cause of the H5N1 HPAI viral infection in these wild birds remained elusive, it is entirely conceivable that mandarin ducks might have been infected by contacting other infected water birds, and Eurasian eagle owls might have been opportunistically infected by ingesting H5N1 HPAI virus-infected birds. Pheasants and mandarin ducks are the most important prey for Eurasian eagle owls living in forested areas in Korea [25]. 

The pathogenicity of the H5N1 HPAI viruses in wild birds is diverse, ranging from asymptomatic to lethal according to the virus strain, host species, host age, viral dose and infection route. In experiments with A/turkey/Turkey/1/2005, which belongs to clade 2.2 [26], tufted ducks (*Aythya fuligula*) and Eurasian pochards (*Aythya ferina*) were severely affected and died, while mallards, common teals (*Anas crecca*), Eurasian wigeons (*Anas Penelope*) and gadwalls (*Anas strepera*) were infected subclinically but excreted the virus through the OP and CL routes. In addition, another clade 2.2 H5N1 HPAI virus (A/chicken/Korea/IS/2006) [27] causes lethal infection in mute swans (*Cygnus olor*) and ruddy shelduck (*Tadorna ferruginea*), but not in greylag geese (*Anser anser*), mandarin ducks and mallards. Although there has been no report yet on the pathogenicity of the clade 2.3.2.1 viruses in wild birds, many species of wild migratory birds (mandarin ducks, Baikal teals, whooper swans, white-fronted geese and spot-billed ducks) became infected and died of clade 2.3.2.1 H5N1 HPAI virus infection during the 2010/2011 winter season in Korea [18]. However, some of the viruses were isolated from a captured healthy mallard duck as well as fecal samples from a mallard duck and mandarin ducks [19,20] collected at the birds’ wintering sites in Korea. These observations also indicate that the pathogenicity of the clade 2.3.2.1 Korean H5N1 HPAI viruses varies in wild bird species, and some wild bird species, such as mallard ducks and mandarin ducks, might act as a vector of the H5N1 HPAI viruses. Elucidation of the exact pathobiology of the viruses in various wild bird species is needed to clarify the causes of high incidences of H5N1 HPAI virus infection in wild birds.

PSC24-24 and EEO/23 viruses displayed high genetic similarity to each other in all eight gene segments (over 99.6%, data not shown), and both of the viruses harbored highly pathogenic characteristics such as multiple basic amino acids at the HA cleavage site (PQRERRRKR/GLF). Additionally, the PSC24-24 and the EEO/23 viruses showed high pathogenicity in chickens (IVPI value of the PSC24-24 and the EEO/23 strains were 2.74 and 2.86, respectively) and all of the chickens inoculated with the EEO/23 intranasally were dead within 3 days. Likewise, in the field, sudden death and high mortality in chicken farms were observed during the 2010/2011 Korean H5N1 HPAI outbreak [18].

When we assessed the pathogenicity of the two Korean H5N1 HPAI viruses in ducks, they produced a lethal infection, and the survival curves showed no statistical significance between the two viruses (*p* = 0.0538, Fig 2). However, the mortality rates were different between PSC24-24 and EEO/23 viruses, at 50% and 100%, respectively. Although the two Korean isolates have a similar genetic background, there were 10 amino acid differences between, and further study is required to elucidate which factor mediated the differences in the mortality rates in the duck inoculation experiment. In virus recovery tests, exuberant shedding of both viruses through the OP and CL routes was evident (Table 3), and PSC24-24 and EEO/23 viruses were detected in all tested tissues (Table 4). Moreover, both viruses were transmitted efficiently to un-inoculated contact ducks, and sero-conversion was observed in all of the survivors (Table 3). 

Korea has experienced four H5N1 HPAI outbreaks caused by different virus genotypes: clade 2.5, 2.2, 2.3.2.1 and 2.3.2.1 in 2003/2004, 2006/2007, 2008 and 2010/2011, respectively. Clade 2.5 (A/chicken/Korea/ES/2003) and clade 2.2 (A/chicken/Korea/IS/2006) viruses can replicate systemically in ducks, however, they proved unlethal to young ducks [15,28]. In contrast, clade 2.3.2.1 virus (A/chicken/Korea/Gimje/2008), isolated in 2008 [29], and the viruses assessed in this study established lethal infections in 2-week-old ducks. These results are comparable to a previous report of the high pathogenicity (100% mortality) of genotypically related clade 2.3.2.1 Mongolian virus (A/whooper swan/Mongolia/6/2009) in 4-week-old domestic ducks [14]. 

Although the clinical outcomes of H5N1 HPAI infection in ducks depends on the virus strains, infection dose, route and host factors such as ages, immune status, genetic backgrounds and so forth, field epidemiological data indicated age-dependent pathogenicity in ducks during the 2010/2011 HPAI outbreaks in Korea. Commercial meat-type ducks < 5-week-of-age displayed sudden death and high mortality; however, the prominent clinical signs of the egg laying breeder ducks were a large decrease in egg production rate (up to 80%) and reduced feed consumption, but rarely death (Table 1). According to the previous reports [30,31], the pathogenicity of some H5N1 HPAI viruses showed age dependence in ducks. Pantin-Jackwood *et al*. [32] elucidated that the differences in the disease severity observed in different aged ducks are in part caused by the differences in innate immune responses, *i.e*. the younger ducks have a less efficient innate immune response to the virus. Song *et al*. [24] proposed that the combination of 224P and 383D in the PA protein of the H5N1 HPAI virus is the virulence marker in ducks and that the specific amino acids have a cumulative effect in pathogenicity in ducks. The two Korean isolates have an aspartic acid (D) on the 383 site but have a serine (S) rather than a proline (P) on the 224 site in the PA protein. The 224S and 383D combination is common in influenza A viruses. In addition, the two Korean isolates have virulence markers for mallard ducks, such as 436Y in PB1 and 515T in the PA protein [33] and 51M, 56V and 87E in PB1-F2 [34]. However, those molecular characteristics are not a unique feature of the two Korean isolates and are shared with previous Korean H5N1 HPAI viruses (A/chicken/Korea/ES/2003, A/chicken/Korea/IS/2006 and A/chicken/Korea/Gimje/2008, data not shown) and other H5N1 HPAI viruses [35]. Therefore, we speculate that there are other mechanisms that determine the virulence of the virus in ducks. To elucidate the age dependence of the virulence of the Korean virus isolates in ducks and explain the circumstances of the field outbreaks in duck farms in the 2010/2011 winter season in Korea, pathogenicity assessments of the isolates in aged ducks are required.

The two Korean H5N1 HPAI viruses in this study do not have reported mammalian virulence markers, such as 627K [36] 701N [37,38] and R714 [39] in the PB2 protein, and S66 in the PB1-F2 [40]. Furthermore, the viruses exhibit 226Q and 228G (H3 numbering) on the RBS of the HA molecule, a motif that has a demonstrated affinity for the avian cellular sialic acid motif [41]. However, the viruses produced severe clinical signs and disease progression (depression, labored breathing and death) in mice. The MLD_50_ of the two isolates were 10^3.4^ (PSC24-24) and 10^3.6^ EID_50_ (EEO/23) (Table 5), meaning that the viruses displayed high pathogenicity in mice, based on the criteria proposed in a previous report [42] in which samples of MLD_50_ under 10^3.8^ EID_50_ were considered to be highly pathogenic, and those over 10^5.5^ EID_50_ were considered to have low pathogenicity in mice. Both PSC24-24 and EEO/23 replicated efficiently in mouse brains and lungs, with high titers ranging from 10^4.8^ to 10^7.7^ TCID_50_/g of tissue without pre-adaptations (Table 5; data converted to viral titer per gram of tissue). Similar results published recently [35] demonstrated that a recent clade 2.3.2.1 H5N1 HPAI virus (A/chicken/Jiangsu/k0402/2010) which does not have such established genetic determinants, showed high pahtogenicity in mice without prior adaptation and the authors speculated that there may be other mechanisms for mouse virulence of the virus. Although there is no evidence that clade 2.3.2.1 genotype H5N1 HPAI viruses have gained the ability to infect non-avian species, especially humans [1], the wide global distribution of clade 2.3.2.1 viruses and their high virulence in non-avian animals have raised concerns about human health.

Although the two Korean H5N1 HPAI viruses originated from different hosts, they showed similar genetic and phylogenetic properties and exhibited high pathogenicity in animal inoculation experiments, including a mammalian animal model (mice). Continuous surveillance to detect new influenza viruses in animals is needed to counter one of the most dangerous diseases in animals as well as in humans.

## 4. Experimental Section

### 4.1. Viruses used in this Study

A/Eurasian eagle owl/Korea/23/2010 (EEO/23) was isolated from a dead Eurasian eagle owl (*Bubo bubo*) rescued by the Chungnam Wild Animal Rescue Center on November 26, 2010. A/Mandarin duck/Korea/PSC24-24/2010 (PSC24-24) was isolated from a fecal sample collected from a wild bird habitat, and the host of the virus was determined to be a mandarin duck (*Aix galericulata*) by a DNA barcoding system, as previously described [43]. Tissues and the fecal samples were suspended in antibiotic-treated phosphate buffered saline (PBS, pH 7.2) and inoculated into 9-11-day-old specific pathogen-free (SPF) embryonated chicken eggs (ECE) (SPAFAS, USA) via the allantoic cavity. Eggs were incubated at 37°C, and virus replication was determined by a hemagglutination assay with 1% chicken red blood cells. 

### 4.2. Sequencing and Phylogenetic Analysis

Sequencing and phylogenetic analysis were performed as described [44]. Briefly, the viral RNA was extracted from allantoic fluids with a Viral Gene-spin Viral DNA/RNA Extraction Kit (Intron Biotechnology, Korea). All eight viral RNA segments were amplified using segment specific primers [45,46] using a OneStep RT-PCR premix kit (Qiagen, USA). The PCR products were purified with a MEGAquick-spin extraction kit (Intron Biotechnology, Korea), and the products were directly sequenced (Macrogen, Korea). The sequences were deposited in the public database [GenBank: JQ710446 – JQ710461]. We used the MEGA 5 program [47] to perform multiple sequence alignments with the Clustal W algorithm and analyzed the phylogenetic relationships between the viruses using the neighbor-joining method and a Kimura-2 parameter model with a bootstrap value of 1000.

### 4.3. Animal Experiments

#### 4.3.1. Chicken inoculation experiments

To elucidate the pathogenicity in chickens, eight 6-week old SPF chickens were inoculated with 1/10 diluted infectious allantoic fluid of PSC24-24 and EEO/23 viruses intravenously for IVPI test according to the OIE (World Organization for Animal Health) protocol and 10^6.0 ^EID_50_/0.1 ml of EEO/23 virus was inoculated to eight chickens intranasally. Tissues from the dead chickens in the EEO/23 virus intranasally inoculated group were aseptically derived, and the virus titers were measured in chicken embryo fibroblast (CEF) cell culture.

#### 4.3.2. Duck inoculation experiments

To assess the pathogenicity in ducks, eight 10-day old commercial Pekin ducks free from antibodies to avian influenza virus were intranasally inoculated (10^6.0 ^EID_50_/0.1 ml) with PSC24-24 or EEO/23 HPAI virus. After 8 h, three ducks in either group were co-housed as a contact group. Among the eight ducks in inoculated group, two ducks per group were sacrificed at 3 day-post-inoculation (dpi) for the elucidation of virus distribution in infected hosts, and various tissue samples were aseptically derived. The tissues were used to prepare 10% (w/v) homogenates in PBS containing antibiotics, and the virus titer was measured in CEF cell culture. 

Virus shedding was determined through oropharyngeal (OP) and cloacal (CL) swabs at 1, 3, 5, 7, 10 and 14 dpi. The samples were inoculated in CEF cell culture, and the virus growth was determined by observing a cytopathic effect (CPE). 

Serum samples from live ducks at 14 dpi were taken and tested with a hemagglutination inhibition (HI) test to detect antibodies to the AIV HA protein using eight hemagglutination units of homologous antigen according to the OIE protocol. 

#### 4.3.3. Mouse inoculation experiments

To determine the mean mouse lethal dose (MLD_50_) and the 50% mouse infectious dose (MID_50_) we intranasally inoculated PSC24-24 and EEO/23 in eight 6-week-old female BALB/c mice (ORIENT BIO, Korea) per group with 10^0.0^ to 10^7.0^ EID_50_/50 μl of 10-fold serially diluted virus under light anesthesia with Zoletil (Virbac S.A., France). For the MID_50_, we took the lung tissues from three euthanized mice per group at 3 dpi, and virus replication was determined in CEF cell culture with tissue homogenates. The remaining five mice per group were observed for 14 days to determine the MLD_50_. To determine the virus replication in mice, three mice from the 10^6.0 ^EID_50_ virus-inoculated group were sacrificed at 3 and 6 dpi, and multiple organs were collected. All animal experiments were performed in a biosafety level 3 facility and with the permission of the Animal Ethics Committee in the Animal, Plant and Fisheries Quarantine and Inspection Agency in Korea.

### 4.4. Statistical Analysis

The ANOVA test for analyzing the mouse body weight changes and a log-rank test for comparing the survival curves in ducks and mice were conducted with a Prism 5 program package (ver. 5.0; GraphPad Software, USA).

## 5. Conclusions

We assessed the genetic and pathobiological characteristics of two Korean H5N1 HPAI viruses isolated from wild birds during the 2010/2011 HPAI outbreak in Korea. One was isolated from a wild duck (mandarin duck), and the other was isolated from a raptor (Eurasian eagle owl). The viruses were phylogenetically classified into the clade 2.3.2.1 genotype, which has been widely distributed in many Asian and European countries and caused numerous deaths in wild birds and tremendous economic losses in the poultry industry. In animal inoculation experiments, both viruses replicated systemically and produced high lethality in chickens, ducks and mice, regardless of the original hosts. These results raise concerns that the clade 2.3.2.1 H5N1 HPAI viruses circulating world-wide in poultry and wild birds might be a threat to public health, and continuous surveillance is needed to prepare for newly appearing influenza viruses.
